# MiR-5195-3p predicts clinical prognosis and represses colorectal cancer progression by targeting TLR4/MyD88 signaling

**DOI:** 10.1186/s13008-024-00133-x

**Published:** 2024-10-10

**Authors:** Yandong Lv, Shuwei Guo, Lingtong Jin, Kai Wang, Yongsheng Li, Haonan Li, Yikang Lu, Hongzhou Liu

**Affiliations:** Department of Colorectal Surgery, Heping Hospital Affiliated to Changzhi Medical College, No.110 Yanan South Road, Luzhou District, 046000 Changzhi City, Shanxi Province P. R. China

**Keywords:** miR-5195-3p, Prognosis, Colorectal cancer, TLR4, MyD88

## Abstract

**Background:**

Recent studies have highlighted the role of miR-5195-3p in suppressing cell growth in various cancers. However, the specific functional impact of miR-5195-3p in colorectal cancer (CRC) remain to be fully clarified. The importance of miR-5195-3p in CRC was evaluated, aiming to uncover its underlying molecular mechanism and identify it as a potential therapeutic target for CRC.

**Results:**

Our research has shown that miR-5195-3p is markedly under-expressed in CRC tissues and cell cultures, with its reduced presence associated with a higher TNM stage, lymphatic invasion, and unfavorable survival outcome. Ectopic miR-5195-3p expression curtailed proliferation, migration, and invasion of SW1116 and HT29 cells. Additionally, we discovered that miR-5195-3p directly targets and negatively influences Toll-like receptor 4 (TLR4) in CRC cells. Moreover, an inverse relationship was noted between miR-5195-3p and TLR4 expression in CRC tissue samples. Notably, restoring TLR4 expression counteracted miR-5195-3p’s suppressive impact on cell growth, motility, invasiveness, epithelial-mesenchymal transition (EMT), and the TLR4/MyD88 signaling pathway in SW1116 and HT29 cells.

**Conclusions:**

MiR-5195-3p suppresses the CRC cellular functions through the downregulation of TLR4/MyD88 signaling, indicating that targeting miR-5195-3p might offer a viable therapeutic strategy for CRC.

**Supplementary Information:**

The online version contains supplementary material available at 10.1186/s13008-024-00133-x.

## Background

Colorectal cancer (CRC) stands as the second most common cause of cancer fatalities, resulting in nearly 1 million deaths annually and representing approximately 10% of all new cancer diagnoses and deaths globally [1]. Despite advancements in treatment through surgical resection, radiotherapy, and chemotherapy that have notably enhanced local and regional management for a majority of CRC patients, challenges such as late detection, distant metastases, and high recurrence rates continue to hinder patient survival [2, 3]. Thus, it is vital to enhance our understanding of the molecular activities associated with aggressive CRC to boost the effectiveness of therapeutic interventions.

MicroRNAs (MiRNAs/miRs), a class of endogenous non-coding RNA sequences comprising 22–25 nucleotides, typically induce the de-adenylation and decay of mRNA, leading to the downregulation of protein synthesis by targeting the 3′-untranslated region (3′-UTR) of specific genes [4, 5]. In the last twenty years, miRNAs have become a focal point of research due to their critical roles in the initiation and progression of various cancers, particularly due to their vital functions in regulating cell proliferation, migration, apoptosis, and metastasis [6–9]. In this context, miR-5195-3p, a relatively unexplored miRNA, has been identified as a significant player across various types of cancer. The study conducted by Jiang et al. [10] suggested that miR-5195-3p hinders the growth and spread of bladder cancer cells by focusing on KLF5. Subsequently, research conducted by Wang and colleagues [11], as well as Yang and their team [12], highlighted miR-5195-3p’s function as a tumor suppressor in osteosarcoma and glioma by specifically targeting NEDD9 and BIRC2, respectively. Moreover, in the case of NSCLC, miR-5195-3p could inhibit cell growth, movement, and invasion through regulating MYO6 expression [13]. The objective of this research is to explore the previously unexamined clinical importance and the role of miR-5195-3p in CRC cells.

Toll-like receptors (TLRs) constitute a broad family of pattern recognition receptors crucial for initiating inflammatory reactions [14], among which TLR4 is a prominent member often found to be overexpressed in several human cancers, including prostate cancer [15] and breast cancer [16]. Upon detecting tumor antigens, TLR4 triggers the activation of MyD88 (myeloid differentiation factor 88), which in turn initiates the translocation of NF-κB to the nucleus, followed by the transcription of target genes [17]. Research indicates that increased expressions of TLR4 and MyD88 are associated with diminished overall survival in ovarian cancer patients [18, 19]. The signaling cascade involving TLR4 and MyD88 is understood to promote the development and spread of breast cancer [20], with its suppression being recognized as a promising treatment strategy for hepatocellular carcinoma [21] and epithelial ovarian cancer [22]. Previous bioinformatic analyses from our research indicated that the seed sequence of miR-5195-3p may have putative binding sites within the 3′-UTRs of the TLR4 gene. Notably, activated TLR4/MyD88 signaling axis has been linked to reduced overall survival [23] and enhanced malignancy in CRC [24]. Drawing from these observations, we propose that the miR-5195-3p/TLR4 axis may be pivotal in governing the biological behaviors of CRC cells.

To substantiate our hypothesis, we began by examining miR-5195-3p and TLR4 expression levels in CRC tissues. We utilized Chi-squared test, Kaplan–Meier method, and Cox regression analysis to examine the clinical significance of miR-5195-3p in CRC patients. Subsequently, we conducted a range of functional tests to explore if miR-5195-3p controls CRC cell growth, movement, invasion, and the EMT process by negatively affecting the TLR4/MyD88 pathway.

## Results

### Downregulation of mir-5195-3p in CRC correlates with poor prognosis

**Fig. 1:**
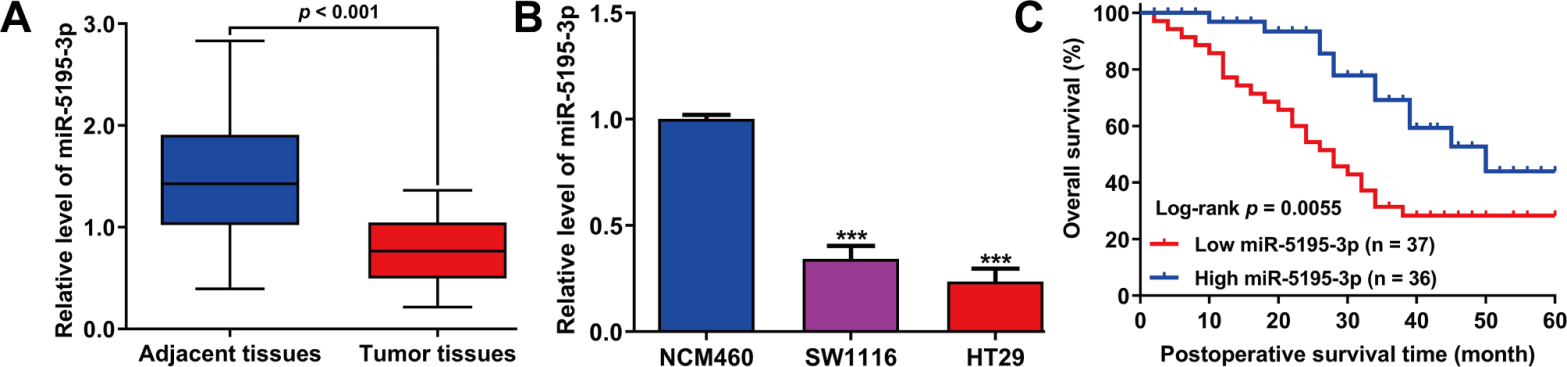
The expression of miR-5195-3p was downregulated in CRC and associated with poor prognosis. (**A**) The expression level of miR-5195-3p was detected by quantitative real time PCR in tumor and adjacent tissues derived from CRC patients. *p* < 0.001, compared with adjacent tissues, t-test; (**B**) The expression level of miR-5195-3p was determined in CRC cell lines (SW1116 and HT29) and normal human colonic epithelial cells NCM460 by quantitative real time PCR. ***p < 0.001, compared with NCM460, one-way ANOVA followed by Dunnett’s test; (**C**) The Kaplan–Meier curves of overall survivals with high and low miR-5195-3p expressions

Quantitative real-time PCR revealed significantly reduced miR-5195-3p expression levels in CRC tumor tissues, when compared with adjacent non-tumor tissues (Fig. 1A). This reduction was also observed in SW1116 and HT29 cells compared to the normal NCM460 cells (Fig. 1B), suggesting a widespread decrease of miR-5195-3p in CRC. For the analysis of clinical outcomes, patients were divided into groups with high and low expression levels of miR-5195-3p in tumor tissues by using the median expression values as the threshold. Chi-squared test showed an association between higher miR-5195-3p levels and TNM stage as well as lymph node invasion (Table 1). In CRC patients, reduced miR-5195-3p levels were correlated with lower overall survival rates (Fig. 1C). Furthermore, miR-5195-3p was confirmed as an independent prognostic factor for overall survival (Table 2).

**Table 1:** Association between Mir-5195-3p expression and clinicopathological characteristics of CRC patients

Characteristics	Cases (*n* = 73)	miR-5195-3p expression level		*P*-value
		Low (*n* = 37)	High (*n* = 36)	
**Age (years)**				0.101
< 60	25	16	9	
≥ 60	48	21	27	
**Gender**				0.295
Female	32	14	18	
Male	41	23	18	
**Tumor size (cm)**				0.562
< 5	49	26	23	
≥ 5	24	11	13	
**TNM stage**				0.003*
I-II	43	28	15	
III-IV	30	9	21	
**Tumor differentiation**				0.939
Good/moderate	51	26	25	
Poor/undifferentiated	22	11	11	
**Lymph node invasion**				0.021*
Negative	48	29	19	
Positive	25	8	17	

*p < 0.05; **Abbreviations**: CRC, colorectal cancer

**Table 2:** Univariate and multivariate analysis for overall survival in CRC patients

	Univariate analysis		Multivariate analysis	
Characteristics	HR (95% CI)	*P* value	HR (95% CI)	*P* value
Age (years)	2.012 (1.203–3.256)	0.465	NA	NA
Gender	0.963 (0.358–1.562)	0.596	NA	NA
Tumor size (cm)	2.314 (0.813–4.385)	0.124	NA	NA
TNM stage	1.263 (0.575–3.475)	0.326	NA	NA
Tumor differentiation	3.015 (1.415–5.426)	0.425	NA	NA
Lymph node invasion	2.975 (0.775–4.659)	0.008*	2.265 (0.694–3.956)	0.023*
miR-5195-3p expression	2.315 (0.745–4.275)	0.023*	1.975 (0.415–3.485)	0.036*

*p < 0.05; **Abbreviations**: CRC, colorectal cancer; HR: hazard ratio; CI: confidence interval; NA, not analyzed

### MiR-5195-3p overexpression inhibited the proliferation, migration, and invasion of CRC cells

**Fig. 2:**
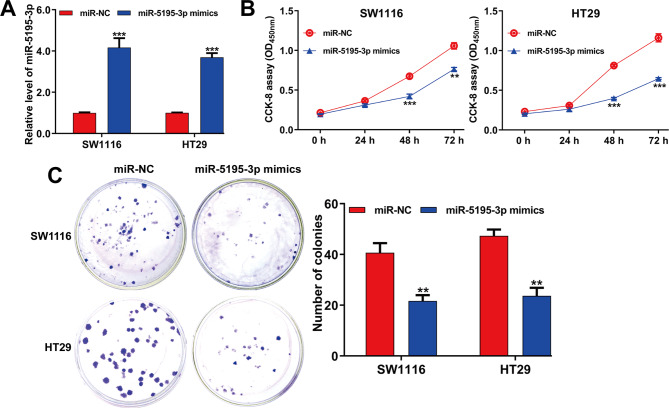
Effects of miR-5195-3p overexpression on CRC cell proliferation. SW1116 and HT29 cells were transfected with miR-5195-3p mimics or miR-NC for 48 h. (**A**) The expression level of miR-5195-3p in transfected SW1116 and HT29 cells was measured by quantitative real time PCR. (**B**) The proliferation curves of transfected SW1116 and HT29 cells were plotted after performing CCK-8 assay. (**C**) Colony formation assay performed in transfected SW1116 and HT29 cells. Left, representative images of colonies. Data were expressed as means ± SD of three independent experiments. **p < 0.01, ***p < 0.001, compared with miR-NC

**Fig. 3:**
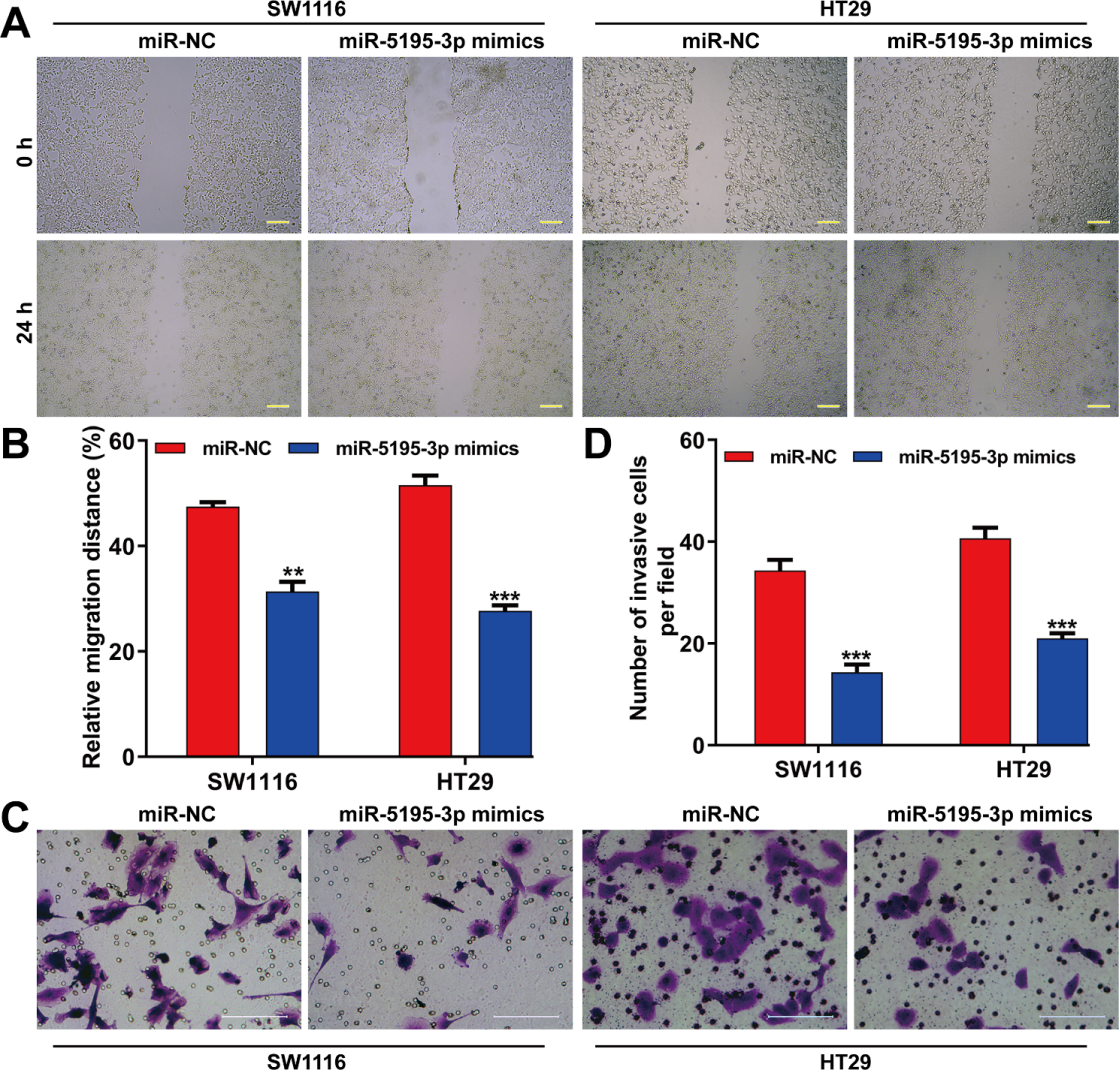
Effects of miR-5195-3p overexpression on CRC cell migration and invasion. (**A**) Representative images of wound healing (×200 amplification) and (**B**) quantification of relative migration distance was shown in transfected SW1116 and HT29 cells. (**C**) Representative images of transfected SW1116 and HT29 cell invasive ability. (**D**) Cell invasiveness was quantified by counting cells that passed through the Matrigel membrane, using a light microscope (×200). Scale bars, 100 μm; Data were expressed as means ± SD of three independent experiments. **p < 0.01, ***p < 0.001, compared with miR-NC

Quantitative real-time PCR results indicated a significant elevation in miR-5195-3p levels in both SW1116 and HT29 cells following transfection with miR-5195-3p mimics (Fig. 2A). Functional assay were conducted to explore miR-5195-3p’s role in CRC cells. The data from CCK-8 assay depicted a significantly impaired cell proliferation rate after miR-5195-3p mimics transfection in SW1116 and HT29 cells (Fig. 2B). Colony formation assays echoed this finding, with a significant decrease in colonies in the miR-5195-3p mimics group (Fig. 2C). Scratch wound healing and transwell assays revealed decreased movement and invasive cells with miR-5195-3p overexpression (Fig. 3A-D), collectively suggesting that miR-5195-3p negatively influences CRC cell functions.

### Identifying TLR4 as a gene targeted by miR-5195-3p

**Fig. 4:**
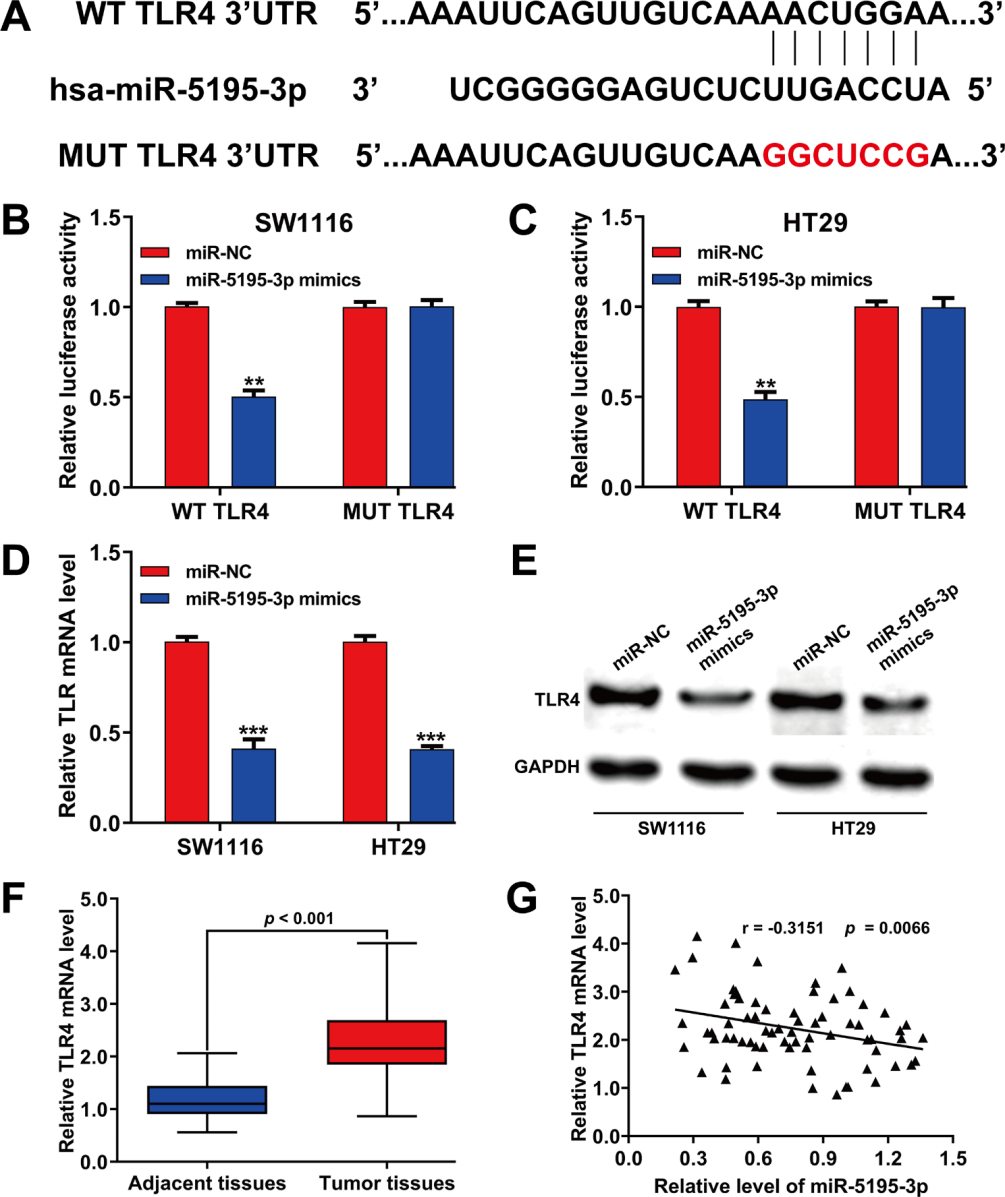
TLR4 as a target gene of miR-5195-3p in CRC cells. (**A**) Schematic of miR-5195-3p putative binding sites in WT 3ʹ UTR of TLR4 and MUT 3′-UTR of TLR4. (**B-C**) Relative luciferase activity of TLR4 3′-UTR in SW1116 and HT29 cells co-transfected with the indicated reporters and miR-5195-3p mimics oligonucleotides or miR-NC. (**D**) Quantitative real time PCR analysis of the mRNA levels of TLR4 in SW1116 and HT29 cells transfected with miR-5195-3p mimics or miR-NC. (**E**) Western blot analysis of the protein levels of TLR4 in SW1116 and HT29 cells transfected with miR-5195-3p mimics or miR-NC. (**F**) The mRNA level of TLR4 was detected by quantitative real time PCR in tumor and adjacent tissues derived from CRC patients. (**G**) Spearman’s analysis of the correlation between miR-5195-3p and mRNA levels of TLR4 mRNA expression in human CRC tissues. Data were expressed as means ± SD of three independent experiments. **p < 0.01, ***p < 0.001, compared with miR-NC

Predictive studies revealed a matching site for miR-5195-3p within the 3′-UTR of TLR4 (Fig. 4A). This direct interaction was verified through luciferase reporter assays, which demonstrated notable decreases in WT TLR4 luciferase activity in SW1116 (Fig. 4B) and HT29 cells (Fig. 4C) after miR-5195-3p overexpression, while mutant plasmids remained unaffected. The TLR4 mRNA (Fig. 4D) and protein expression **(**Fig. 4E) were both significantly decreased in cells with miR-5195-3p overexpression, indicating direct interaction with TLR4’s 3ʹ-UTR in CRC cells. Additionally, TLR4 mRNA levels were notably higher in CRC samples than in adjacent tissues (Fig. 4F). A negative correlation between miR-5195-3p and TLR4 mRNA levels was also observed in CRC specimens (*r* = − 0.3151, *p* = 0.0066, Fig. 4G).

### TLR4 overexpression counteracts miR-5195-3p’s inhibitory effects on CRC cells

**Fig. 5:**
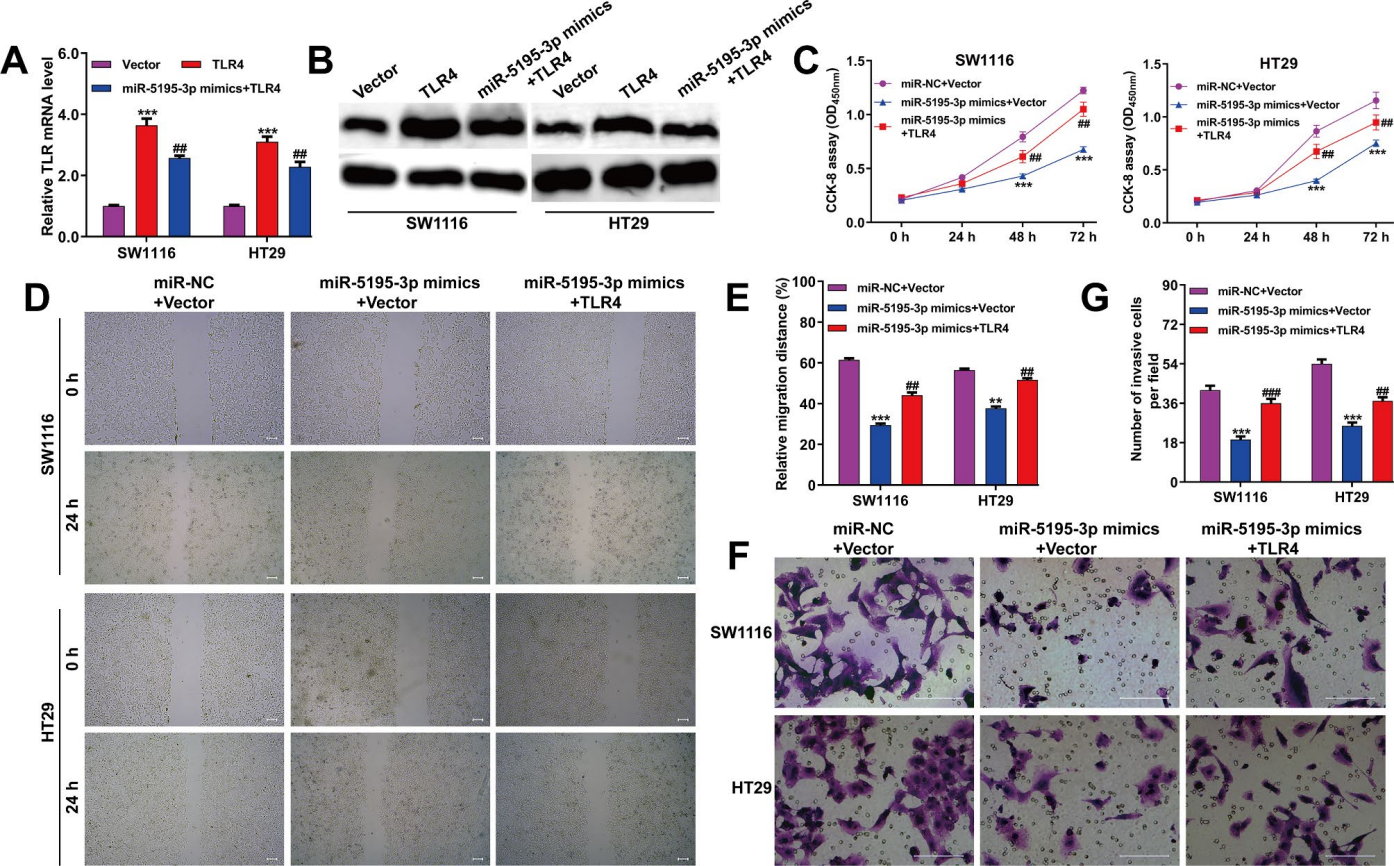
TLR4 overexpression can rescue miR-5195-3p mimics-mediated inhibition of proliferation, migration and invasion in CRC cells. (**A**) Quantitative real time PCR analysis of TLR4 mRNA expression in SW1116 and HT29 cells co-transfected with miR-5195-3p mimics and TLR4 plasmid. (**B**) Western blot analysis of TLR4 protein expression in SW1116 and HT29 cells co-transfected with miR-5195-3p mimics and TLR4 plasmid. The effects of co-transfection of miR-5195-3p mimics and TLR4 plasmid on proliferation were determined using CCK-8 assay (**C**), on migration using wound healing assay (**D-E**), and on invasion using Matrigel-coated Transwell assay (**F-G**) in SW1116 and HT29 cells. Scale bars, 100 μm; Data were expressed as means ± SD of three independent experiments. **p < 0.01, ***p < 0.001, compared with Vector or miR-NC + Vector; ##*p* < 0.01, ###*p* < 0.001, compared with TLR4 or miR-5195-3p mimics + Vector

**Fig. 6:**
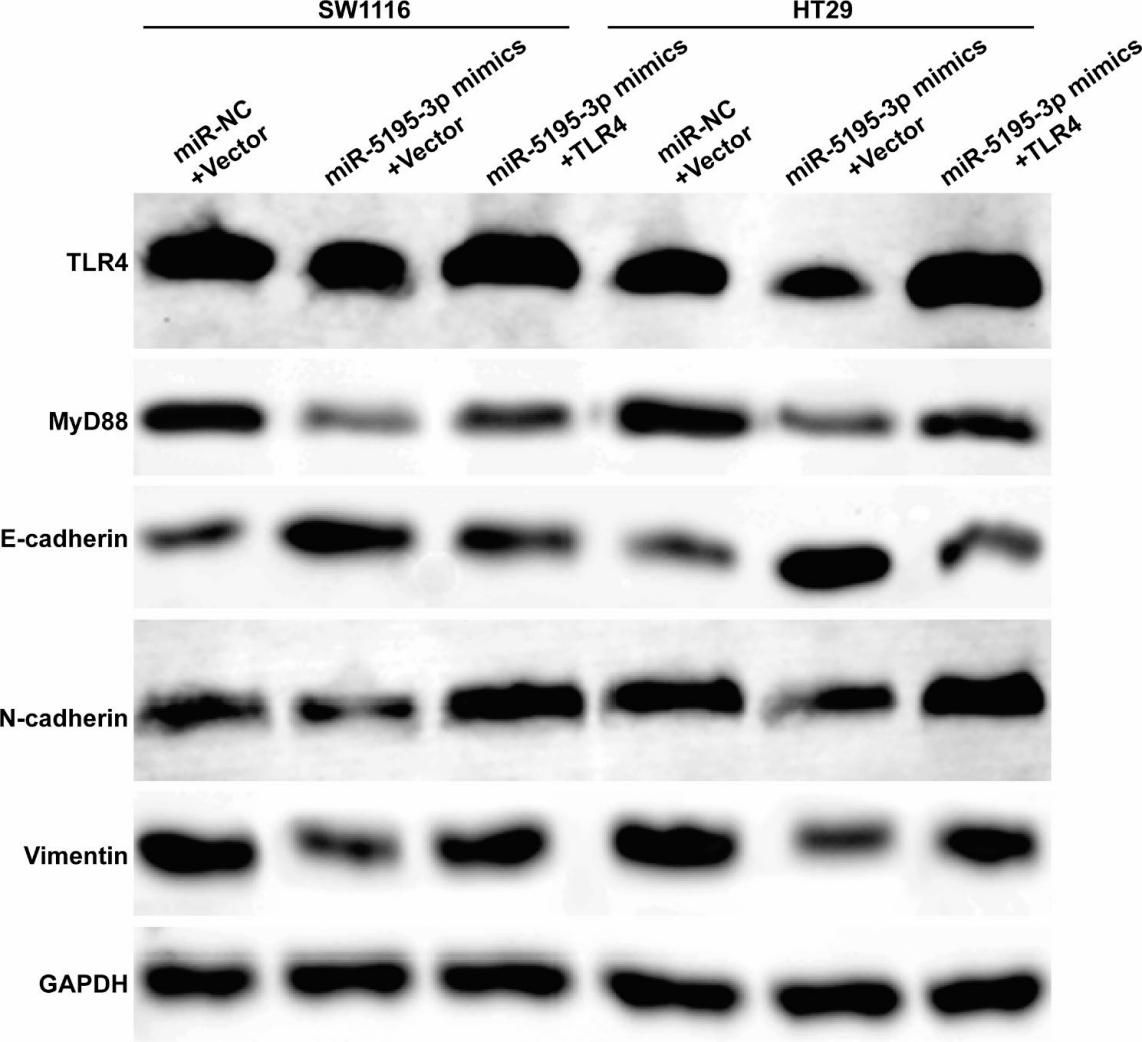
TLR4 overexpression reversed the effects of miR-5195-3p on TLR4/MyD88 signaling-mediated EMT process. Western blot analysis was performed to measure the protein levels of TLR4, MyD88, E-cadherin, N-cadherin, and Vimentin in SW1116 and HT29 cells co-transfected with miR-5195-3p mimics and TLR4 plasmid

To assess TLR4’s role in miR-5195-3p’s effects on CRC cells, SW1116 and HT29 cells underwent co-transfection with TLR4 plasmids and miR-5195-3p mimics. Figure 5A-B illustrated that miR-5195-3p mimics co-transfection diminished the increase in TLR4 mRNA and protein levels due to TLR4 plasmid transfection. Functional tests revealed that TLR4 overexpression could counteract miR-5195-3p mimics-mediated suppressive effects on these cell lines, as confirmed by the CCK-8 assay (Fig. 5C), scratch wound healing (Fig. 5D-E), and transwell assays (Fig. 5F-G). In parallel, miR-5195-3p overexpression caused reduced levels of TLR4/MyD88 signaling markers (TLR4 and MyD88), N-cadherin, and Vimentin, but an increase in E-cadherin. However, these alterations were negated upon TLR4 overexpression (Fig. 6). This indicates that the suppressive influence of miR-5195-3p on CRC cells may predominantly operate through the downregulation of TLR4.

## Discussion

Numerous studies have highlighted abnormal miRNA expression in CRC, noting a reduction in tumor suppressor miRNAs and an increase in oncogenic miRNAs [25]. In the current study, we noted a marked decrease in miR-5195-3p levels in both CRC tissues and cell lines. Additionally, miR-5195-3p has emerged as a key independent predictor of overall survival in CRC patients. This trend of reduced miR-5195-3p expression mirrors findings in NSCLC [13, 26], hepatocellular carcinoma [27], and ovarian cancer [28]. Zeng et al. [29] linked lower miR-5195-3p levels to higher Gleason scores, advanced TNM stages, and poorer survival outcomes in prostate cancer. Similarly, Wang et al. [30] correlated decreased miR-5195-3p expression with higher FIGO stages, greater invasion depth, and poorer survival prognosis in ovarian cancer patients.

We then demonstrated that miR-5195-3p overexpression significantly inhibited SW1116 and HT29 cell proliferation, migration, and invasion. This was accompanied by alterations in EMT-related proteins, including an elevation in E-cadherin and reduction in N-cadherin and Vimentin in the miR-5195-3p overexpression groups. EMT is crucial in CRC metastasis, involving a transformation from an epithelial to a mesenchymal phenotype, characterized by morphological changes, diminished cell polarity, lower levels of cell-cell adhesion, and increased abilities to migrate and invade, thereby facilitating metastasis [31, 32]. Essential markers of EMT are crucial for enhancing migration and invasion during cancer metastasis, including the downregulation of E-cadherin and the upregulation of Vimentin [33]. Likewise, Yang et al. [13] discovered that elevating miR-5195-3p levels in NSCLC cells diminished their proliferation, migration, and invasion capabilities. Moreover, research indicates miR-5195-3p’s ability to restrain growth and boost chemosensitivity in triple-negative breast cancer [34], as well as its role in curbing proliferation and EMT, and enhancing apoptosis in NSCLC by focusing on VEGFA [26]. Based on this evidence, it is possible that miR-5195-3p downregulates transcription factors that promote EMT, such as N-cadherin and Vimentin. By suppressing these factors, miR-5195-3p can prevent the transition of epithelial cells to a mesenchymal phenotype, which is associated with increased migratory and invasive abilities in CRC cells. Furthermore, our study, which incorporated more CRC cell lines and functional assays than Jahangiri Moez et al. [35], highlights miR-5195-3p as a critical modulator of EMT in CRC.

Bioinformatics analysis pinpointed the TLR4 gene as likely targeted by miR-5195-3p, revealing TLR4 mRNA levels were negatively correlated with miR-5195-3p levels in CRC tissues. A rescue experiment showed that TLR4 overexpression could counteract the tumor-inhibitory roles of miR-5195-3p in CRC cell behaviors by enhancing TLR4/MyD88 signaling. Previous studies have highlighted TLR4’s crucial role in tumor growth regulation in breast cancer and its correlation with cancer growth and metastasis [36]. TLR4 is known to promote metastasis in various cancers by influencing the EMT process [37, 38], and its interaction with MyD88 is linked to increased tumor development and progression in intestinal tumorigenesis [39]. Wang et al. [23] found TLR4/MyD88 signaling overexpressed in CRC, affirming its oncogenic role. Bates et al. [40] linked TLR4-MyD88 signaling to paclitaxel resistance. TLR4 is also targeted by tumor suppressor miRNAs like miR-216a in renal cell carcinoma [41], miR-145-5p in melanoma [42] and miR-122 in hepatocellular carcinoma [43], paralleling the miR-5195-3p/TLR4 axis in CRC. As upstream regulatory mechanisms controlling miR-5195-3p expression, such as ST8SIA6 in hepatocellular carcinoma [27], circ_0009910 in acute myeloid leukemia [44], and circRUNX1 in lung adenocarcinoma [45] have been reported. These will be our next research goals in future work.

This study demonstrates that miR-5195-3p notably diminishes the CRC cell proliferation and migration, potentially by repressing the EMT process through TLR/MyD88 signaling. This reveals miR-5195-3p as a potential therapeutic candidate and prognostic indicator for CRC. However, to comprehensively grasp miR-5195-3p’s role in CRC, additional research is needed, including in vivo studies and investigations into the upstream and downstream signaling mechanisms of the miR-5195-3p/TLR4 axis.

## Materials and methods

### Clinical samples and cell cultures

From January 2017 to December 2022, a total of 73 CRC patients contributed both tumor and matched non-tumor tissue specimens (situated a minimum of 5 cm away from the tumor margin) for examination by a pathologist. None had prior radiotherapy or chemotherapy. The research received approval from the Ethics Committee of Heping Hospital Affiliated to Changzhi Medical College (Shanxi, China), which was conducted in compliance with the Declaration of Helsinki. All participants signed their informed consent.

Acquisition of two human CRC cell lines, SW1116 and HT29, as well as the normal colonic epithelial NCM460 cells, was facilitated through the ATCC (Manassas, VA, USA). These cell cultures were propagated using DMEM (Gibco, Grand Island, New York), with an addition of 10% FBS (Gibco), and were incubated under a 5% CO_2_ atmosphere at 37 °C.

### Cell transfection

MiR-5195-3p mimics, negative control (miR-NC), TLR4 overexpression plasmid (pcDNA3.1-TLR4), along with corresponding empty vectors, were sourced from GenePharma Co. Ltd. (Shanghai, China). SW1116 and HT29 cells were seeded in six-well plates and subsequently underwent transfection with above oligonucleotides for a duration of 48 h utilized Lipofectamine 2000 (Invitrogen).

### Quantitative real-time PCR

Total RNA was extracted utilizing TRIzol reagent (Invitrogen) and subsequently, reverse transcription was performed with PrimeScript RT reagent (Takara, Dalian, China), strictly adhering to the accompanying guidelines. Quantitative real-time PCR was performed with SYBR Green PCR master mix (Takara) with the following primers: miR-5195-3p (forward: 5′-TAGCAGACTCTTATGATG-3′, reverse: 5′-TGGTGGAGTCGTCGTG-3′); TLR4 (forward: 5′-ATGCCAGGATGATGTCTGCC-3′, reverse: 5′-GGGAGGTTGTCGGGGATTTT-3′); U6 (forward: 5′-CTCGCTTCGGCAGCACA-3′, reverse: 5′-AACGCTTCACGAATTTGCGT-3′) and GAPDH (forward: 5′-GGTGAAGGTCGGAGTCAACG-3′, reverse: 5′-GCATCGCCCCACTTGATTTT-3′). PCR assays were conducted three times, utilizing the 2^−ΔΔCT^ approach to standardize the expression of miR-5195-3p and TLR4 relative to U6 and GAPDH, accordingly.

### CCK-8 assay

Post-seeding into 96-well plates (3,000 cells/well), cells were incubated with 5% CO_2_ at 37 °C. After cultures reached the 24, 48, and 72-h marks, we added 10 µl of CCK-8 reagent (Sigma) to each well and continued to incubate for 2 h. Subsequently, we measured the optical density (OD) at 450 nm, allowing for the plotting of cell proliferation curves based on absorbance.

### Colony formation assay

After seeded into six-well plates (500 cells/well), cells underwent a two-week cultivation period. Post-culture, colonies were rinsed with PBS, followed by 4% paraformaldehyde fixing and 0.5% crystal violet staining. Colonies with more than 50 cells were subsequently examined and tallied under a microscope.

### Scratch wound-healing assay

A scratch wound-healing assay was conducted to evaluate the migratory potential of CRC cells. Initially, cells were cultured in six-well plates (5 × 10^5^ cells/well) until 90% confluence. Then, a 10 µl pipette tip was utilized to made artificial scratches across the cell monolayer, marking the initial scratch width (W0). Post a 24-h incubation, images were taken to document the healing, specifically recording the scratch width at 24 h (W24) under 200× magnification (Olympus IX81, Tokyo, Japan). The formula for calculating relative migration distance is: (W0 - W24)/W0 × 100%.

### Transwell invasion assay

The cell invasive capabilities were evaluated through a transwell assay, utilizing chambers coated with 8 μm pore size Matrigel (Corning, Lowell, USA). Total 100 µl of serum-free medium containing 2 × 10^4^ cells, were placed into the upper chamber, while the lower chamber received 500 µl 10% FBS-enriched medium. Post a 24-h incubation period, the migrated cells to the lower chamber were stained using 1% crystal violet. Subsequently, the invasive cells were examined and counted across five fields chosen at random under a microscope.

### Bioinformatic prediction and luciferase reporter assay

We used the TargetScan7.1 database to forecast target genes of miR-5195-3p and pinpoint TLR4. Then, we cloned both wild-type (WT) and mutant-type (MUT) variants of the TLR4 3′-UTR targeted by miR-5195-3p into the psiCHECK-2 vector (Promega, Madison, WI, USA), generating luciferase reporter plasmids for WT and MUT TLR4. Using Lipofectamine 2000, we transfected SW1116 and HT29 cells with these plasmids, in conjunction with miR-5195-3p mimics or miR-NC. At 48 h after transfection, the Dual-Luciferase Reporter Assay System (Promega) was employed to ascertain firefly and renilla luciferase activities.

### Western blot analysis

After isolation of protein samples and quantification via a BCA assay kit, we performed protein separation via 10% SDS-PAGE and then transferred them onto PVDF membranes. At room temperature, the membranes were first blocked for 2 h using 5% fat-free milk, followed by an overnight incubation with primary antibodies against TLR4, E-cadherin, N-cadherin, MyD88, Vimentin, and GAPDH at 4 °C. After three washes with PBS, the membranes underwent a 2-h incubation at room temperature with HRP-linked secondary antibodies. Protein signals were then identified with an enhanced chemiluminescence kit, with GAPDH serving as the internal control.

### Statistical analysis

Statistical evaluation was performed with GraphPad Prism 8.0 and presenting data as mean ± SD. Using the Chi-squared test, we analyzed the association between miR-5195-3p expression and clinicopathological characteristics. Survival curves were produced through the Kaplan–Meier method and examined using the log-rank test. Prognostic factors for overall survival were identified through Cox regression analysis. The correlation between miR-5195-3p and TLR4 was determined by Pearson’s coefficient. For comparing groups, Student’s t-test and one-way ANOVA, were applied, deeming *p* < 0.05 as significant.

## Electronic supplementary material

Below is the link to the electronic supplementary material.

Supplementary Material 1

## Data Availability

No datasets were generated or analysed during the current study.
