# Supplementary figures and images for: MiR-5195-3p predicts clinical prognosis and represses colorectal cancer progression by targeting TLR4/MyD88 signaling
Source: Cell Div. 2024 Oct 10;19:29. doi: 10.1186/s13008-024-00133-x (PMC11468180; doi:10.1186/s13008-024-00133-x)

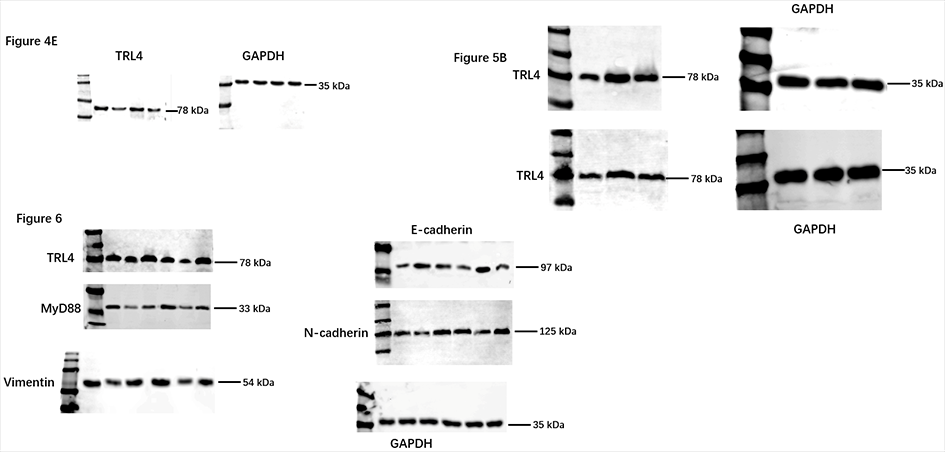

Supplement: Supplementary file 1 — Supplementary Material 1 [file 13008_2024_133_MOESM1_ESM.tif]
